# Metabolomics-Guided Identification of a Distinctive Hepatocellular Carcinoma Signature

**DOI:** 10.3390/cancers15123232

**Published:** 2023-06-18

**Authors:** Vincent Tambay, Valérie-Ann Raymond, Corentine Goossens, Louise Rousseau, Simon Turcotte, Marc Bilodeau

**Affiliations:** 1 Laboratoire d’Hépatologie Cellulaire, Centre de Recherche du Centre Hospitalier de l’Université de Montréal, Montréal, QC H2X0A9, Canada; vincent.tambay@umontreal.ca (V.T.); valerie-ann.raymond.chum@ssss.gouv.qc.ca (V.-A.R.); corentine.goossens@gmail.com (C.G.); 2 Biobanque et Base de Données Hépatobiliaire et Pancréatique, Centre Hospitalier de l’Université de Montréal, Montréal, QC H2X0C1, Canada; louise.rousseau.chum@ssss.gouv.qc.ca (L.R.); simon.turcotte.med@ssss.gouv.qc.ca (S.T.); 3 Département de Chirurgie, Service de Transplantation Hépatique et de Chirurgie Hépatobiliaire et Pancréatique, Centre Hospitalier de l’Université de Montréal, Montréal, QC H2X0C1, Canada; 4 Département de Médecine, Université de Montréal, Montréal, QC H3T1J4, Canada

**Keywords:** liver, hepatocellular carcinoma, metabolic reprogramming, metabolomics, liquid chromatography–mass spectrometry, NMR spectroscopy, metabolites

## Abstract

**Simple Summary:**

Hepatocellular carcinoma is the third most prevalent cancer world-wide. This study aimed to reveal the metabolic signature of hepatocellular carcinoma compared to adjacent normal liver cells. To achieve this, metabolites were detected, analyzed, and quantified using targeted and non-targeted metabolomics. We found distinct metabolite signatures between both sample types. Targeted metabolomics identified distinct metabolites being specifically altered in hepatocellular tissue compared to adjacent liver, supporting the concept of metabolic reprogramming in hepatocellular carcinoma.

**Abstract:**

Background: Hepatocellular carcinoma (HCC) is a major contributor to cancer-related morbidity and mortality burdens globally. Given the fundamental metabolic activity of hepatocytes within the liver, hepatocarcinogenesis is bound to be characterized by alterations in metabolite profiles as a manifestation of metabolic reprogramming. Methods: HCC and adjacent non-tumoral liver specimens were obtained from patients after HCC resection. Global patterns in tissue metabolites were identified using non-targeted ^1^H Nuclear Magnetic Resonance (^1^H-NMR) spectroscopy whereas specific metabolites were quantified using targeted liquid chromatography–mass spectrometry (LC/MS). Results: Principal component analysis (PCA) within our ^1^H-NMR dataset identified a principal component (PC) one of 53.3%, along which the two sample groups were distinctively clustered. Univariate analysis of tissue specimens identified more than 150 metabolites significantly altered in HCC compared to non-tumoral liver. For LC/MS, PCA identified a PC1 of 45.2%, along which samples from HCC tissues and non-tumoral tissues were clearly separated. Supervised analysis (PLS–DA) identified decreases in tissue glutathione, succinate, glycerol-3-phosphate, alanine, malate, and AMP as the most important contributors to the metabolomic signature of HCC by LC/MS. Conclusions: Together, ^1^H-NMR and LC/MS metabolomics have the capacity to distinguish HCC from non-tumoral liver. The characterization of such distinct profiles of metabolite abundances underscores the major metabolic alterations that result from hepatocarcinogenesis.

## 1. Introduction

Cancer is a complex disease characterized by the occurrence of a panoply of cellular alterations. Indeed, the neoplastic transformation of cells has been described by 10 hallmarks, which are attributable to genetic alterations and cellular adaptations to the tumor environment [1,2,3]. Metabolic reprogramming has become of major interest in cancer cell biology. Cancer cells require extensive modifications of cell metabolism to survive and proliferate in an array of environmental conditions [2]. Metabolic reprogramming has also been shown to be highly dynamic during carcinogenesis and cancer development, for example during the epithelial–mesenchymal transition (EMT) within the metastatic cascade [4,5]. The reprogramming of cell metabolism occurring in cancer cells encompasses all modifications of biosynthetic and bioenergetic pathways that allow sustained survival, optimal proliferation, as well as invasion and metastasis. Consequently, the fundamental implication of cell metabolism in the onset and progression of malignancy questions whether cancer is a metabolic disease.

Among the most deadly and prevalent malignancies world-wide is hepatocellular carcinoma (HCC) [6]. HCC is the most frequent primary liver cancer with a median five-year survival rate of approximately 20% [6]. This results mainly from the lack of strategies for early detection combined with limited curative therapeutic options, in addition to its association with chronic liver disease. Since the liver acts as the heart for systemic metabolism [7,8], it comes as no surprise that metabolic alterations are observed in the setting of HCC. Indeed, whereas normal hepatocytes are programmed to maintain normal metabolic homeostasis for the whole body, HCC cells need to maximize the availability of nutrients and metabolic substrates for their optimal growth and survival.

The Warburg effect is among the most widely accepted and studied metabolic phenomena in cancer cell metabolism. In the 1920s, Otto Warburg observed that cancer cells metabolized glucose through glycolysis and lactic acid fermentation rather than the mitochondrial pathway [9]. Since then, major studies have demonstrated the importance of metabolic plasticity and heterogeneity in cancer. For example, the avidity for exogenous glucose in HCC cells has been linked with tumorigenic potential in mice [10]. Lipid metabolism has also been shown to exhibit major alterations in cancer, where certain tumor types have enhanced free fatty acid uptake that has in turn been linked to tumor aggressiveness [11]. Understanding mitochondrial dysfunction is also of major interest in defining the deregulation of cancer cell energetics. In HCC, major modifications of the key enzymes involved in the tricarboxylic acid (TCA) cycle have been reported [12]. Mitochondrial fission within cancer cells has also been associated with an increase in the expression of lipogenic genes, with a concomitant decrease in fatty acid oxidation (FAO) genes [13]. Furthermore, alterations in mtDNA due to reactive oxygen species (ROS) accumulation have been proposed as the cause of mitochondrial dysfunction in hepatocarcinoma cells [14]. Distinct metabolic profiles have also been observed between tumors having high EMT activity compared to those with low EMT activity [15]. For example, in adrenocortical carcinoma, high levels of intratumor nucleotides correlate with enhanced EMT activity [15].

The origin of metabolic alterations in cancer cells is thought to arise from genetic alterations, including those that induce oncogenic signaling, as well as from adaptations to the cancer cell microenvironment. Hypoxia is a phenomenon not unknown to tumors: the rapid growth of cells frequently overcomes their vascular network, resulting in limited oxygen and nutrient availability [16]. Hypoxia-inducible factors (HIF) are transcription factors that respond to oxygen levels and act as major regulators of cell metabolism in addition to certain signaling pathways, including that of TGF-β through PI3K/Akt/mTOR [11]. The stabilization of HIF is thought to be key in promoting angiogenesis, invasion, and metastasis [16]. Under hypoxia, HIF1 has been shown to suppress fatty acid oxidation within mitochondria leading to a decrease in the burden of ROS accumulation from mitochondrial metabolism [17]. Nutrient availability has also been shown to impact the metabolic program of cancer cells: HCC cells have been shown to rewire energy metabolism toward FA oxidation under glucose deprivation [10]. Interestingly, in murine HCC, obesity has been linked with a net decrease in FA oxidation within HCC tumors with a resulting increase in dependence on glucose and glutamine in oxidative phosphorylation [18]. Furthermore, the downregulation of carnitine palmatoyltransferase II (CPT2) in HCC has been shown to limit lipotoxicity from microenvironments characterized by excessive lipids and thus allow cancer cell growth [18].

Moreover, distinct genetic alterations have distinct consequences on cancer cell metabolism. For example, p53, a major tumor suppressor often mutated in cancer cells, is an important regulator of glycolysis and glucose transporters [11]. p53 mutations have been shown to promote aberrant lipid metabolism by inducing sterol regulatory element binding protein (SREBP) activity [11]. Mutations of the PTEN tumor suppressor, which is linked to aberrant Akt activity and thus glucose uptake and metabolism, induce SREBP and subsequent lipid metabolism in cancer cells [11]. On the other hand, fundamental oncogenes have been associated with metabolic reprogramming in cancers. Namely, c-Myc, Kras, and mutations of EGFR have been linked with aberrant glycolysis, glutaminolysis, amino acid metabolism, and pentose phosphate pathway activities [11]. Oncogenic Ras and Src signaling have also been shown to promote normoxic activation of HIF1 by inhibiting prolyl hydroxylase domain (PHD) proteins, which could at least partially explain the development of anaerobic metabolism in the presence of oxygen [16]. In HCC, β-catenin mutations have been shown to drive CPT2 expression and an increase in FAO [17]. Additionally, transcriptomics of HCC tissues has identified molecular patterns of metabolism-related gene expression, which have even proposed metabolism-based molecular classifications of HCC [19,20,21]. Altogether, the current state of the literature suggests that metabolism is intimately linked with cancer onset and progression.

Hence, metabolomics has become a compelling novel tool for understanding cancer cell biology. Metabolomics has major potential in identifying novel clinical biomarkers for the screening, diagnosis, and monitoring of cancers and could aid in identifying cancer risk factors as well as developing novel metabolism-focused targeted therapies [22,23]. Indeed, metabolomics generates robust and highly specific metabolic information, which makes them potentially very useful in the context of personalized cancer medicine [24]. This study aimed to identify the metabolomic signatures of HCC by highlighting key changes in the metabolism of hepatocarcinoma compared to adjacent non-tumoral liver tissue as well as calling attention to the pertinence of metabolic reprogramming in HCC. To achieve this, both targeted and non-targeted metabolomics modalities were used in order to offer a comprehensive understanding of HCC metabolomics. Whereas ^1^H-Nuclear Magnetic Resonance (^1^H-NMR)-based non-targeted analyses allowed the identification of global patterns within the metabolite profiles of non-tumoral and neoplastic liver tissues, liquid chromatography/mass spectrometry (LC/MS)-based targeted analyses enabled the quantification of a specific set of metabolite alterations.

## 2. Materials and Methods

### 2.1. Patients and Sample Collection

Patients (*n* = 5) undergoing HCC tumor resection were recruited with written informed consent prior to surgery. For each participant, one specimen of tumoral tissue was collected and snap-frozen in liquid nitrogen, accompanied by the collection of one specimen of non-tumoral (normal) liver tissue at distance from the neoplastic foci. The time elapsed between the collection of samples and their cryopreservation is shown in Table S1. This research protocol was conducted in accordance with the Declaration of Helsinki and was approved by the “Comité d’éthique de la recherche du Centre de recherche du CHUM (CRCHUM)”. All studied HCC tumors presented a trabecular pattern within livers devoid of underlying cirrhosis. Available clinical data are reported in Table 1.

### 2.2. Metabolite Extraction for Targeted LC/MS-Based Metabolomics

Water-soluble metabolites were extracted from tissue specimens using liquid–liquid extraction. Samples were homogenized in ice-cold metabolite extraction buffer (80% methanol, 2 mM ammonium acetate, pH 9.0; 20 µM [13C10,15N5]-AMP as an internal standard) using a Cryolis-cooled Precellys 24 Dual system (Bertin, France) with CK14 ceramic beads, for 2 × 25 s at 6000 rpm separated by a 15-second rest. Homogenates were centrifuged at 20,000× *g* for 10 min (4 °C); 183 µL of supernatant was transferred to 10 × 75 mm glass tubes and diluted with 367 µL of extraction buffer. Diluted supernatants were mixed and incubated on ice for 10 min. Then, 250 µL of water and 880 µL of chloroform:heptane (3:1) solution were added and samples were mixed thoroughly, followed by a 15-minute incubation on ice. Sample preparations were then centrifuged for 15 min at 4500× *g* (4 °C) and 500 µL of the aqueous/upper phase was transferred to polypropylene tubes for the concentration of extracted metabolites. Organic solvents were removed in a refrigerated CentriVap (Labconco, Kansas City, MO, USA; 90 min, 10 °C) and the remaining liquid (100 µL) was freeze-dried overnight. Prior to LC/MS processing and analysis, 40 µL of water was added to each sample followed by rapid 5-minute centrifugation (4 °C); re-suspended concentrated metabolite extracts were transferred to HPLC vials.

### 2.3. Metabolite Extraction for Non-Targeted ^1^H-NMR-Based Metabolomics

For ^1^H-NMR metabolomic profiling, extraction of liver specimen metabolites was performed by dual-phase methanol:water:chloroform (2:1:2) extraction. Tumor and non-tumoral specimens were ground and homogenized in 600 µL of extraction buffer (2:1 methanol:water) with ceramic beads in a Precellys Homogenizer (Bertin Technologies, Montigny-le-Bretonneux, France). Then, 400 µL of chloroform was added to each sample, followed by a 15-minute incubation (4 °C). Samples were centrifuged for 15 min at 15,000× *g* (4 °C). The upper polar phase was collected and dried overnight under nitrogen flow. Prior to ^1^H-NMR processing and analysis, dried tissue extracts were dissolved in 600 µL of D2O-prepared phosphate buffer solution (pH 7.4) containing 0.4 mM of sodium trimethylsilyl-(2,2,3,3-d_4_)-propionate (TSP) as an internal reference, and re-suspended samples transferred to 5 mm NMR tubes.

### 2.4. Targeted LC/MS Metabolite Detection and Data Acquisition

Liver specimen metabolites and standard analyte solutions were separated by liquid phase chromatography using a Nexera X2 Ultra-High-Performance Liquid Chromatography (UHPLC) system (Shimadzu, Kyoto, Japan) at 40 °C with 3 µL injections on a Poroshell 120 EC-C18 2.1 mm × 75 mm × 2.7 µm UHPLC column (Agilent Technologies, Santa Clara, CA, USA) following a Poroshell 120 EC-C18 2.1 mm × 5 mm × 2.7 µm UHPLC guard column (Agilent Technologies, USA). To perform this, gradient elution with an initial mobile phase was used, consisting of 95%: 10 mM tributylamine and 15 mM acetic acid in water, pH 5.2; 5%: acetonitrile:water (95:5, *v*/*v*) fortified with 0.1% formic acid; this was performed at a flow rate of 0.75 mL/min. Standard and sample metabolites were detected using negative electrospray ionization on a SCIEX 4000 Qtrap mass spectrometer (Framingham, MA, USA). MS/MS parameters were optimized for each metabolite and quantified using SCIEX MultiQuant 3.0.2 (Framingham, USA) according to calibration curves (0.15 to 12,000 pmol per injection) of pure analytes purchased from Sigma Aldrich (Oakville, ON, Canada), prepared in water. Values were normalized per mg of tissue.

### 2.5. Non-Targeted ^1^H-NMR Metabolite Detection and Data Acquisition

The detection of liver sample metabolites for ^1^H-NMR analysis was performed on an Ascend 700 MHz spectrometer (Bruker, Billerica, MA, USA) coupled to an AVANCE NEO console equipped with a 5-millimeter triple resonance probe (Bruker, USA) at 298 K. For each sample, a one-dimensional ^1^H-NMR spectrum was acquired with water peak suppression using a nuclear Overhauser enhancement spectroscopy (NOESY) presaturation pulse sequence; 128 scans; 65,000 data points; an acquisition time of 2.4 s; a relaxation delay of 4 s; a mixing time of 10 milliseconds; and a spectral width of 20 ppm.

### 2.6. ^1^H-NMR Spectral Processing and Analysis

After acquisition of ^1^H-NMR spectra for each specimen, free induction decays were multiplied by an exponential function equivalent to a 0.3 Hz line-broadening factor before applying Fourier transform. The spectra were phased and the baseline was corrected and referenced to the TSP peak (at 0 ppm) using TopSpin 4.0.5 (Bruker, USA). One-dimensional spectra ranging from 0.5 to 9.5 ppm were binned by intelligent adaptive bucketing and the corresponding spectral areas were integrated using the NMRProcFlow tool (https://www.nmrprocflow.org/; accessed on 18 February 2020). The spectral region from 4.5 to 5 ppm was removed as this corresponded to residual water within samples. Total spectral areas were calculated using the remaining buckets and followed by constant sum normalization.

### 2.7. Statistical Analysis

Various statistical methods were used to analyze metabolomic datasets from LC/MS and ^1^H-NMR metabolite profiling modalities. For both datasets, all samples were normalized according to each original specimen’s wet weight, then metabolites/variables were mean-centered and divided by the square root of their standard deviation (Pareto scaling) prior to subsequent statistical analysis. To obtain a reduced dimensionality view of the metabolomic profiles of HCC and non-tumoral liver samples, principal component analysis (PCA) and partial least squares–discriminate analysis (PLS–DA) were performed using MetaboAnalyst 5.0 (https://metaboanalyst.ca/; accessed on 2 September 2022). PCA allowed the identification of global trends in metabolite signatures between study groups and of clusters and possible outliers within the metabolic data matrices in an unsupervised analytical manner. As a supervised statistical analysis, PLS–DA allowed the maximization of the covariance between the observed abundances of metabolites in liver samples and the sample type (tumoral and non-tumoral samples). In PLS–DA, the number of components was determined by “Leave One Out Cross-Validation” which yielded goodness-of-fit (R_2_) and predictability (Q_2_) of the regression. Loadings plots obtained from PLS–DA allowed the identification of metabolites greatly contributing to the metabolomic discrimination of liver samples between both study groups. The most weighted metabolites in this statistical classification of the analyzed samples were identified using the variable importance in projection (VIP) method. Student’s *t* test was used to measure statistical differences in specific metabolite concentrations between both sample groups. Statistical differences were considered significant when *p* < 0.05.

## 3. Results

### 3.1. ^1^H-NMR Profiling Identifies a Distinct Metabolomic Signature of HCC

**Figure 1:**
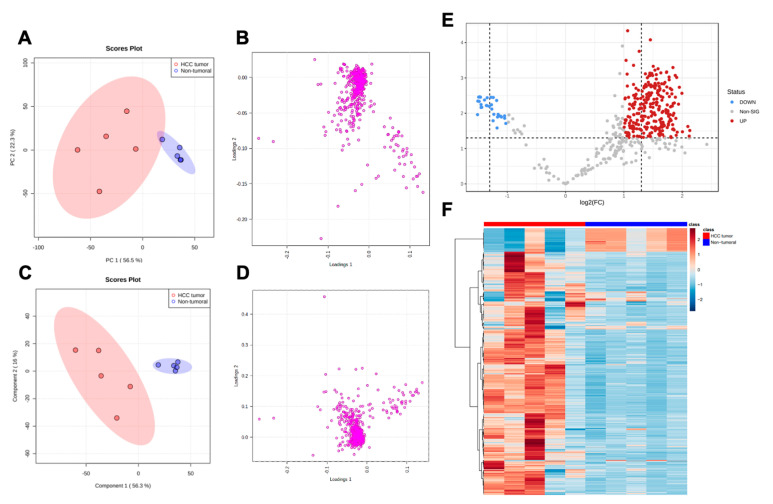
^1^H-NMR profiling of HCC compared to paired non-tumoral liver tissue. Non-targeted metabolomics was performed by ^1^H-NMR after metabolite extraction. Processed spectra and binning with intelligent adaptive bucketing of 450 bins (metabolomic features) were integrated and analyzed with principal component analysis (PCA): PCA scores plot (**A**), PCA loadings plot (**B**). Metabolomic profiles between both sample groups were compared using partial least squares–discriminate analysis (PLS–DA): PLS–DA scores plot (**C**), PLS–DA loadings plot (**D**). Volcano scatter plot (**E**) of significantly altered (*p* < 0.05) features (increased, red; decreased, blue; unchanged, gray) in HCC specimens compared to non-tumoral specimens. Heatmap depiction (**F**) of the differential abundance of the 450 analyzed spectral features in all samples (red, HCC; blue, non-tumoral liver).

To establish the metabolite makeup of HCC, we compared the global metabolomic profile of all liver specimens (tumoral and non-tumoral) using non-targeted ^1^H-NMR. Through intelligent processing methodologies, a total of 450 metabolomic features were analyzed and subsequently compared between all samples. To reduce the dimensionality of the ^1^H-NMR dataset and identify major trends in metabolomic feature abundance between samples, an initial Principal Component Analysis (PCA) was performed (Figure 1A,B). The PCA identified a primary principal component (PC1) explaining 56.5% of metabolic data variability within the 10 studied samples and a second principal component (PC2) explaining 22.3% of data variability (Figure 1A). Interestingly, the identification of individual samples (red: HCC tumors; blue: non-tumoral livers; shaded area: 95% confidence interval for each group) within the PCA scores plot revealed a distinct clustering of both groups along the primary principal component. The PCA loading plot represents all analyzed metabolomic features and their importance in positioning samples within the scores plot. As seen in Figure 1B, a select population of features corresponded to those whose abundances were characteristic of non-tumoral tissue, whereas the abundance of a more important proportion of liver metabolites was characteristic of HCC tumors. Observations made through PCA were further studied using PLS–DA to identify the metabolic discrimination of both sample groups using their respective metabolomic datasets (Figure 1C,D). PLS–DA cross-validation for one component revealed validation metrics with a goodness-of-fit of 0.855 and a model predictability of 0.716 (accuracy = 1.0) and, for two components, revealed a goodness-of-fit of 0.927 and a model predictability of 0.623 (accuracy = 0.9). In this supervised analysis of liver specimen metabolomic profiles, which maximizes covariance between the observed changes in metabolomic features and both study groups, the primary component explained 56.3% of data variability, whereas the second component explained 16.0% (Figure 1C). The PLS–DA loading plot represents all analyzed metabolomic features and their importance in positioning samples within the PLS–DA scores plot (Figure 1D). Interestingly, for both PCA and PLS–DA scores plots, non-tumoral liver samples were circumscribed within a relatively small region of the scores plots, whereas HCC tumor samples were much sparser within the plots and their 95% confidence interval region (Figure 1B,D). In Figure 1E, a volcano scatter plot shows the identified metabolomic features being significantly altered, either increased (red) or decreased (blue), in HCC compared to non-tumoral samples. Features that remained unaltered in hepatocarcinoma samples are identified in gray. For the visualization of the global metabolomic pattern of each studied sample, Figure 1F depicts a heatmap of the relative abundance of all 450 analyzed metabolite features. Clearly, this depiction shows that the identification of the metabolomic signatures of HCC tumors is completely distinct from those of non-tumoral liver.

### 3.2. Targeted HCC Metabolomics Identify Altered Amino Acid and TCA Cycle Profiles

**Figure 2:**
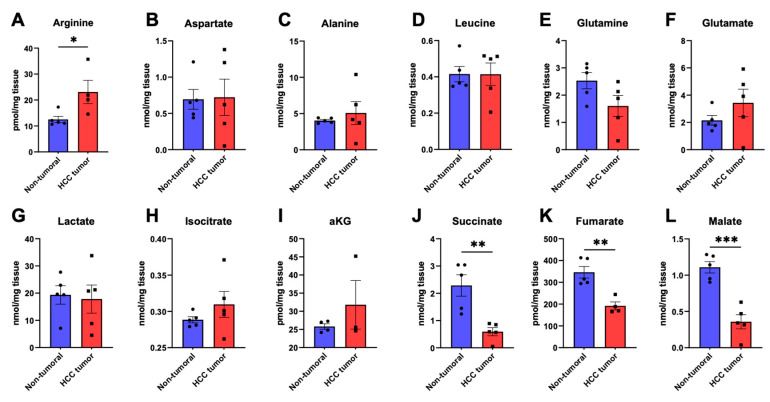
Targeted identification of amino acids and TCA cycle metabolites in hepatocellular carcinoma specimens and adjacent normal liver. HCC (red) and non-tumoral liver (blue) specimens were analyzed through targeted LC/MS metabolomics to characterize the tissue abundances of arginine (**A**), aspartate (**B**), alanine (**C**), leucine (**D**), glutamine (**E**), and glutamate (**F**) amino acids, as well as lactate (**G**), (iso)citrate (**H**), α-ketoglutarate (αKG, (**I**)), succinate (**J**), fumarate (**K**), and malate (**L**). *: *p* < 0.05, **: *p* < 0.01, ***: *p* < 0.001.

Our initial non-targeted analysis of liver tissue metabolomics revealed distinctive metabolite signatures between HCC and non-tumoral liver tissues. This interesting finding, which showcased the capacity of liver metabolomics to successfully discriminate between HCC and non-tumoral liver through metabolite profiling, prompted us to study changes in the tissue abundance of specific metabolites; hence, 26 metabolites from distinct metabolic pathways were chosen for targeted screening of liver specimen metabolomics. Firstly, diverse amino acids were quantified in all samples having been analyzed through ^1^H-NMR profiling. The non-essential amino acid arginine, an important intermediate of the urea cycle, was the only significantly altered amino acid between the two groups. Indeed, the concentration of arginine was higher in HCC (23.1 ± 4.5 pmol/mg_tissue_) compared to non-tumoral specimens (12.5 ± 1.2 pmol/mg_tissue_) (*p* < 0.05, Figure 2A). As seen in Figure 2B–D, no changes in aspartate, alanine, or leucine concentrations were observed in HCC tissues compared to their paired non-tumoral samples. The amino acid glutamine was marginally decreased in HCC specimens (Figure 2E), which was accompanied by an increase in glutamate abundance in certain tumor samples (Figure 2F), though not reaching statistical significance. In addition, we quantified lactate, the product of pyruvate fermentation following glycolysis: its tissue abundance was unchanged in tumors compared to control specimens (Figure 2G). Five intermediates of the tricarboxylic acid (TCA) cycle pathway were also measured in each sample to unveil potential disturbances in mitochondrial metabolism in hepatocarcinoma. The levels of (iso)citrate (Figure 2H) and α-ketoglutarate (Figure 2I) remained similar between HCC and non-tumoral tissue samples. On the other hand, succinate was significantly decreased from 2.29 ± 0.39 nmol/mg_tissue_ in controls to 0.59 ± 0.15 nmol/mg_tissue_ in HCC samples (*p* < 0.01, Figure 2J). Fumarate, another TCA cycle intermediate, was lower in tumors (191.9 ± 18.2 pmol/mg_tissue_) in comparison to non-tumoral specimens (346.2 ± 26.7 pmol/mg_tissue_) (*p* < 0.01, Figure 2K). A similar change was observed for malate, the level of which was greatly reduced in HCC (1.11 ± 0.078 nmol/mg_tissue_) compared to surrounding liver tissue (0.36 ± 0.097 nmol/mg_tissue_, *p* < 0.001, Figure 2L).

### 3.3. Metabolic Reprogramming of HCC Encompasses Major Changes in Energy Metabolism and the Glycerol-3-Phosphate/Dihydroxyacetone Phosphate Pathway

**Figure 3:**
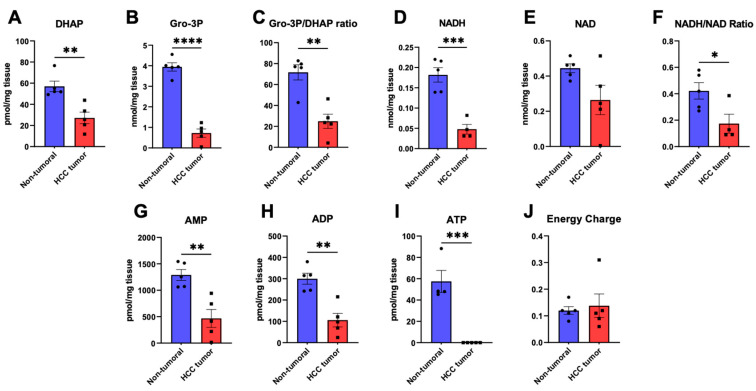
Metabolites and the glycerol-3-phosphate/dihydroxyacetone phosphate pathway in hepatocellular carcinoma specimens and adjacent normal liver. HCC (red) and non-tumoral (blue) specimens were analyzed through targeted LC/MS metabolomics to characterize the tissue abundances of dihydroxyacetone phosphate (DHAP, (**A**)), glycerol-3-phosphate (Gro-3P, (**B**)), and the resulting Gro-3P/DHAP ratio (**C**), NADH (**D**), NAD (**E**), the NADH/NAD ratio (**F**), AMP (**G**), ADP (**H**), and ATP (**I**). The adenylate energy charge was calculated as follows: [ATP + 1/2ADP]/[ATP + ADP + AMP] (**J**). *: *p* < 0.05, **: *p* < 0.01, ***: *p* < 0.001, ****: *p* < 0.0001.

We then studied key metabolic intermediates at the crossroad between glycolysis and lipid metabolism such as dihydroxyacetone phosphate (DHAP) and glycerol-3-phosphate (glycerol-3P) as well as those involved in energy metabolism, such as ATP and the NADH cofactor. Interestingly, both DHAP and glycerol-3P were significantly lower in HCC samples compared to adjacent non-tumoral tissues (Figure 3A,B). Taken together, the calculated ratio of glycerol-3P-to-DHAP was significantly lower in HCC compared to non-tumoral samples (24.9 ± 6.7 vs. 71.7 ± 7.3, respectively; *p* < 0.01, Figure 3C). The concentration of NADH was found to be 0.18 ± 0.018 nmol/mg_tissue_ in non-tumoral specimens and 0.048 ± 0.012 nmol/mg_tissue_ in HCC specimens, which represents a 3.75-fold decrease in HCC samples (*p* < 0.001, Figure 3D). Though the oxidized form of NADH, NAD (Figure 3E), was only marginally lower in tumors, the calculated NADH/NAD ratio (Figure 3F) was significantly lower in HCC (*p* < 0.05). Given this important change in NADH, we studied ATP and its metabolites ADP and AMP to further understand the state of energy storage and metabolism in HCC. As shown in Figure 3G, AMP was significantly lower in HCC (467.0 ± 168.9 pmol/mg_tissue_) compared to non-tumoral liver (1289.5 ± 103.8 pmol/mg_tissue_, *p* < 0.01). A similar trend was observed for ADP, which was nearly three-fold lower (105.6 ± 31.8 pmol/mg_tissue_) in HCC samples (*p* < 0.01, Figure 3H). Importantly, as shown in Figure 3I, ATP could not be detected (0.00 ± 0.00 pmol/mg_tissue_) in HCC tumors in opposition to non-tumoral liver specimens (57.5 ± 10.3 pmol/mg_tissue_) (*p* < 0.001). Finally, the calculated energy charge remained similar between both groups (Figure 3J).

### 3.4. Perturbations of Oxidative-Stress-Related Metabolites Glutathione and NADPH in Hepatocarcinoma Compared to Adjacent Non-Tumoral Tissue

**Figure 4:**
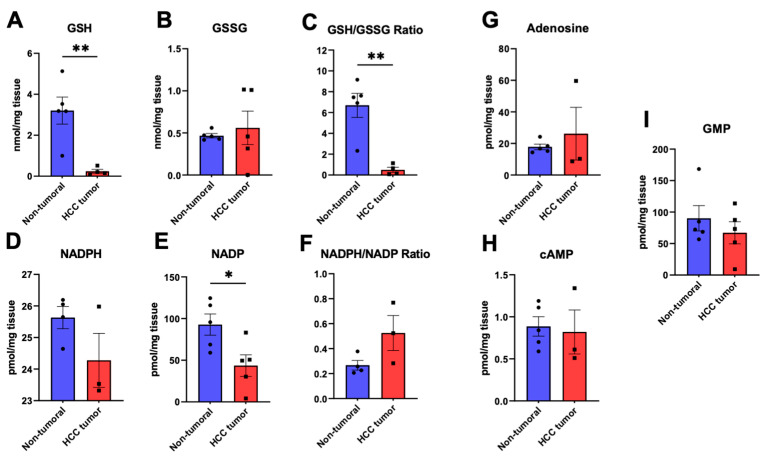
Oxidative stress-related metabolites glutathione and NADPH in hepatocellular carcinoma specimens and adjacent normal liver. HCC (red) and non-tumoral (blue) specimens were analyzed through targeted LC/MS metabolomics to characterize the tissue abundances of reduced glutathione (GSH) (**A**), oxidized glutathione (GSSG) (**B**), the GSH/GSSG ratio (**C**), NADPH (**D**), NADP (**E**), the resulting NADPH/NADP ratio (**F**), as well as adenosine (**G**), cyclic AMP (cAMP), (**H**), and GMP (**I**). *: *p* < 0.05, **: *p* < 0.01.

Oxidative stress is thought to be a key component contributing to tumorigenesis as well as cancer progression. As such, we quantified glutathione, an important mediator of cellular redox homeostasis, in all liver specimens. The reduced form of glutathione, GSH, had a concentration of 3.20 ± 0.66 nmol/mg_tissue_ in non-tumoral specimens and was significantly depleted in HCC tumors (0.24 ± 0.10 nmol/mg_tissue_, *p* < 0.01, Figure 4A). Though the oxidized form of glutathione, GSSG, was relatively unchanged in HCC (Figure 4B), the calculated GSH/GSSG ratio plummeted 13.4-fold from 6.69 ± 1.15 in the non-tumoral liver to 0.50 ± 0.25 in HCC (*p* < 0.01, Figure 4C). Additionally, as glutathione recycling from GSSG to GSH requires NADPH, we quantified the abundance of the latter as well as its oxidized form NADP. NADPH marginally decreased in HCC (Figure 4D) whereas NADP, as depicted in Figure 4E, was significantly lower in HCC tumors (*p* < 0.05). Nonetheless, the NADPH/NADP ratio increased in tumors, though without reaching statistical significance (Figure 4F). Adenosine, cAMP, and GMP abundances remained similar between both study groups (Figure 4G–I). Altogether, targeted LC/MS metabolomics allowed the identification of major changes in the tissue abundance of diverse metabolic intermediates in HCC, supporting the global metabolite signature of hepatocarcinoma having been identified using non-targeted ^1^H-NMR profiling.

### 3.5. Ability of Targeted LC/MS to Characterize HCC and Metabolites Contributing to the HCC Metabolomic Signature

**Figure 5:**
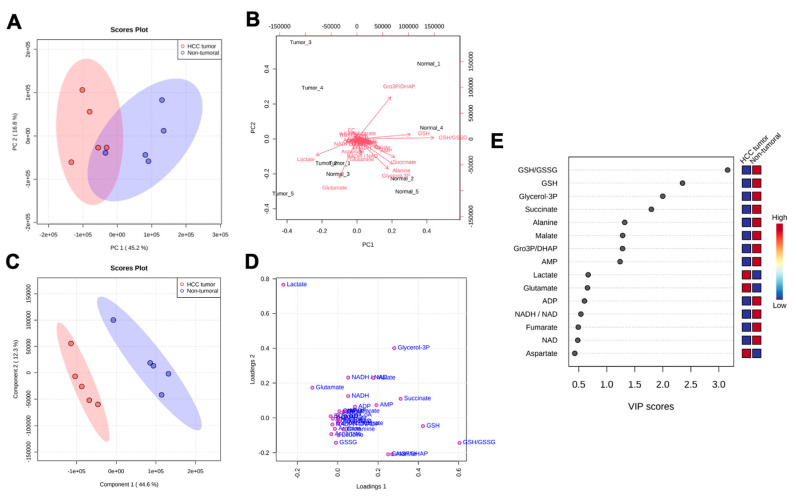
Ability of targeted LC/MS liver metabolomics to discriminate hepatocellular carcinoma specimens and adjacent normal liver. Targeted metabolomics of HCC and paired non-tumoral liver specimens were performed by LC/MS after metabolite extraction for a set of 26 chosen metabolites. Metabolomic profiles of liver specimens were compared using the specific tissue abundance (nmol/mg_tissue_) of all metabolites. Metabolomic profiles were analyzed using principal component analysis (PCA): PCA scores plot (**A**), PCA scores plot with identification of metabolite positioning (**B**). Metabolomic profiles between both sample groups were compared using partial least squares–discriminate analysis (PLS–DA): PLS–DA scores plot (**C**), PLS–DA loadings plot (**D**), and attributed variable importance in projection (VIP) scores (**E**) to important metabolites in the PLS–DA model. Relative abundance of the important metabolites was classified as increased (red) or decreased (blue) in HCC samples compared to non-tumoral liver tissue.

Given the identification of significant differences in the abundance of the many metabolites from various metabolic pathways between HCC and non-tumoral tissue, as observed in Figure 2, Figure 3 and Figure 4, we analyzed our LC/MS metabolomics dataset using multivariate and descriptive statistics. The metabolomic profiles of all studied samples were analyzed through PCA, as shown in the scores plot in Figure 5A. Using the 26 quantified metabolites, unsupervised PCA identified a primary principal component (PC1) that explained 45.2% of metabolite quantification variability between the ten liver specimens; a second observed principal component (PC2) encompassed 16.8% of metabolomic data variability. Then, the labeling of positioned samples within the PCA scores plot according to their respective groups (red: HCC tumors; blue: non-tumoral liver; shaded area: 95% confidence interval for each group) allowed the identification of two well-segregated clusters along the primary principal component (PC1) that corresponded to both study groups (HCC and non-tumoral samples). Further decomposition of the PCA revealed that lactate and glutamate tended to be more abundant in HCC tumor samples, whereas higher levels of GSH, succinate, alanine, glycerol-3P, and AMP were rather distinctive of non-tumoral specimens (Figure 5B). Furthermore, we analyzed our LC/MS dataset using supervised PLS–DA (Figure 5C–E). PLS–DA cross-validation for one component revealed validation metrics with a goodness-of-fit of 0.827 and a model predictability of 0.669 (accuracy = 0.9) and revealed a goodness-of-fit of 0.968 and a model predictability of 0.707 (accuracy = 0.9) for two components. As shown in the scores plot of Figure 5C, PLS–DA component analysis revealed a component one explaining 44.6% and a component two explaining 12.3% of the observed variability of liver metabolomics. Given that PLS–DA is optimized to maximize the relationship between the observed variance of metabolite quantities and the descriptor sample group (tumor vs. non-tumoral), samples were clearly separated without any overlap between both groups along component one of the scores plot (Figure 5C). All studied metabolites and calculated metabolite ratios were depicted in a loadings plot (Figure 5D). In addition, as shown in Figure 5E, PLS–DA attributed variable importance in projection (VIP) scores to all studied features. Metabolites and ratios having a VIP greater than 1.000 were considered significant contributors to the metabolomic discrimination of liver specimens as either belonging to the HCC or the non-tumoral group. Namely, the decreased abundance of GSH, glycerol-3P, succinate, alanine, malate, and AMP, as well as the decrease in GSH/GSSG and glycerol-3P/DHAP ratios were the most discriminant features of the HCC metabolomic signature. The GSH/GSSG ratio had a VIP score of 3.155, and those of GSH, glycerol-3P, succinate, alanine, malate, the glycerol-3P/DHAP ratio, and AMP were 2.351, 1.998, 1.795, 1.319, 1.282, 1.281, and 1.236, respectively.

## 4. Discussion

The phenomenon of altered metabolism within cancer cells emerged nearly one century ago. Nevertheless, much mystery still remains concerning the implication of metabolic reprogramming in cancer pathophysiology from onset to progression. Indeed, changes in biosynthetic metabolism and bioenergetics within cancer cells have yet to be demonstrated effective for cancer management, especially for HCC. From a metabolic point of view, the detection and treatment of neoplastic liver lesions are hindered by the important metabolic function of the normal liver. Targeting metabolism in HCC will need to be specific to pathways preferentially expressed in liver cancer cells and preferably absent or of low importance in functional, normal hepatocytes. The quest for discovering such metabolic targets for anti-neoplastic treatment begins with a better understanding of HCC pathophysiology. Although some studies have reported metabolomics analyses of fluids from HCC patients, the metabolomics of HCC tissue itself has yet to be better characterized and validated [25,26,27]. Nevertheless, metabolomics has become a crucial tool for characterizing cancer cell metabolic behavior [22,28]. This study characterized the metabolomic profiles of human HCC compared to adjacent non-tumoral liver tissue through the identification of metabolite signatures from liver specimens. To achieve this, complimentary metabolomics techniques were performed: non-targeted ^1^H-NMR profiling detected all extracted tissue metabolites within samples whereas targeted metabolomics, through LC/MS, quantified the abundance of a specific ensemble of metabolites.

Our initial analysis of liver tissue metabolomics was performed through a non-targeted approach using five HCC samples and their paired non-tumoral adjacent tissues. The ^1^H-NMR dataset enabled the detection of 450 metabolomic features, many of which were altered in HCC compared to adjacent liver tissue. Indeed, PCA, which aimed to reduce data dimensionality, revealed a PC1 explaining 56.5% of data variability. Further identification of samples in the PCA plot showed that the clusters of both groups, that is HCC or non-tumoral tissues, were clearly separated along the PC1 axis. This suggests that the main factor explaining changes in the abundance of all liver tissue metabolites is attributed to the type of tissue (HCC vs. non-tumoral). Similar observations were made using supervised PLS–DA statistical analyses. HCC and adjacent non-tumoral tissues also showed distinct profiles of metabolomic heterogeneity. Whereas non-tumoral liver samples were well circumscribed within a smaller 95% confidence interval region, samples belonging to the HCC group were scattered within a large 95% confidence interval region. These findings highlight the significance of inter-individual heterogeneity in HCC, which seems to be accordingly much greater than in normal livers [29,30]. Indeed, tumor heterogeneity is an important concept in cancer biology, and our untargeted metabolomics calls attention to cell metabolism as a key component of inter-tumoral heterogeneity in HCC; therefore, untargeted metabolomics has a powerful ability to discriminate whether a given sample is neoplastic or not. PCA of the targeted metabolomics dataset, which quantified 26 specific metabolites within the studied samples, revealed in turn a PC1 explaining 45.2% of data variability. This result is interesting, as specifically measuring only 26 metabolites within liver tissue, rather than detecting all metabolites, had a discriminative capacity nearly matching that of ^1^H-NMR profiling. Indeed, both groups, HCC and adjacent non-tumoral tissues, were separated along the PC1, confirming with non-targeted metabolomics that metabolomic variations are a major hallmark of HCC. Distinct clustering of both groups was also clear along component one of the PLS–DA. Altogether, these findings highlight that changes in the metabolic program of hepatocytes occurring during hepatocarcinogenesis are so important that the study of the metabolite landscape within liver tissue is powerful enough to identify HCC. This discriminative ability of metabolomics has also been described by various groups in lung cancer compared to chronic obstructive pulmonary disease and pancreatic cancer compared to pancreatitis [31,32,33]. Furthermore, Kowalczyk et al. also discussed the ability of specific metabolites to precisely discriminate between subtypes of lung cancer [34].

Additionally, the heatmap depiction and volcano scatter plot of the 450 detected metabolomic features represented in Figure 1 highlight the presence of distinct metabolite patterns between non-tumoral and HCC tissues; hence, a specific population of metabolites is abundant in the normal liver whereas a large population of different metabolites tend to accumulate in HCC. Moreover, according to our targeted metabolomics analysis, the most significant alterations observed between the two groups were lower levels of specific metabolites in HCC. This is different to what was observed in ^1^H-NMR, where many detected metabolites had increased levels in tumors: this could be explained by the fact that the chosen metabolites in the LC/MS targeted approach are indeed those that characterized the population of metabolomic features that specifically decreased in HCC tumors shown by ^1^H-NMR. The important proportion of metabolites that were shown to be increased in HCC tumors compared to non-tumoral tissues by ^1^H-NMR could potentially be waste products and metabolic by-products, which could emerge from rapid and likely inefficient metabolism in cancer cells [35]. Regarding the identity of the metabolites within this population, the profiling of liver sample metabolomic profiles using an initial non-targeted ^1^H-NMR approach aimed to establish global changes in metabolite signatures between the two study groups, that is non-tumoral liver and HCC tumors, rather than specifically identify the detected metabolites within the dataset. Ulterior identification of such metabolite populations could reveal additional metabolic intermediates that belong to the class of oncometabolites, that is, metabolites that are either specific to cancer cells or are greater in abundance within tumoral tissues. An important example of such a phenomenon is the oncometabolite 2-hydroxyglutarate, which is abundant in isocitrate dehydrogenase-mutant cancers including glioma and acute myeloid leukemia [22,36].

Furthermore, our targeted metabolomics approach allowed the quantification of specific metabolites from an array of pathways central to cell and energy metabolism. First, arginine was the only significantly altered amino acid found in HCC, in which its abundance increased. This could possibly be explained should the urea cycle metabolism be shown to be decreased within HCC tumors, which is known to be highly functional in the normal liver [7]. Arginine is the final substrate of the urea cycle, its breakdown by arginase leading the release of urea and ornithine. As such, decreased urea cycle activity within HCC cells, in addition to increased arginine consumption, are plausible explanations for this increase in tissue arginine within tumors compared to adjacent tissue. Interestingly, contrary to the increase in arginine tissue abundance found in HCC, arginine levels have been shown to be decreased in the sera of HCC patients [37]. Together with metabolomic data from Morine et al. and He et al., our findings are complementary in highlighting the important metabolic changes occurring in HCC from the identification of metabolites in liver samples [26,27]. Indeed, our study not only shows that HCC metabolomics has a distinct profile to non-tumoral liver, as suggested in other studies, but ^1^H-NMR analysis has also proven that non-cirrhotic liver exhibits very limited metabolomic variability between individuals, highlighting the importance of studying liver tissue metabolomics in HCC among other liver diseases.

Concentrations of important metabolic intermediates of the TCA cycle, such as succinate, fumarate, and malate, were found to be significantly lower in HCC samples compared to adjacent non-tumoral tissue. This finding could be linked with various aspects of hepatocarcinogenesis. Indeed, this decrease could be explained by the increased turnover of TCA cycle metabolites within HCC cells, namely those exhibiting oxidative metabolism. This turnover can in turn support biosynthetic demands for the genesis of lipids, proteins, and nucleic acids, as well as cellular energy. Another explanation of this interesting finding could be that HCC tumors exhibit increased hypoxic features, hypoxia being a well-known characteristic of cancer. As such, the presence of hypoxia as well as mitochondrial dysfunction within HCC cells could yield a decrease in the flux of cytosolic carbons through mitochondria and the TCA cycle. Though there exists insufficient evidence to support severe hypoxia in HCC, altered oxygen availability is bound to occur in HCC cells when tumor expansion surpasses its inherently irregular angiogenetic program [38,39]. Mitochondrial dysfunction, on the other hand, has been suggested to occur in HCC as a result of mtDNA mutations and copy number variations [14,40]. In a similar perspective, decreased glycerol-3-phosphate and DHAP could be explained by such a phenomenon. Indeed, decreased glycerol-3-phosphate shuttle activity and, as such, decreased flux of high energy electrons toward mitochondria, could be explained by increased tissue hypoxia in HCC tumors. Additionally, DHAP and glycerol-3-phosphate can be used as important bioenergetic and biosynthetic precursors, for example, lipid synthesis within cancer cells, which could explain their decreased abundance in tumors compared to adjacent liver. Nevertheless, an important finding of our targeted metabolomic analysis of HCC and adjacent liver tissues suggests an imbalance in the TCA cycle as a hallmark of the metabolic landscape of hepatocarcinoma. Interestingly, various studies have suggested succinate as an oncometabolite in other tumor types such as paraganglioma, pheochromocytoma, and renal cell carcinoma [41,42,43]. On the other hand, our current metabolomic analysis does not identify succinate, another important TCA cycle intermediate, as a significant oncometabolite in HCC as its abundance statistically decreases within the studied cancer tissues. In fact, the decrease in the abundance of succinate was among the most important features of the metabolomic profile of HCC according to the VIP analysis of the LC/MS dataset. This observation remains to be explained.

Our targeted metabolomic analysis also revealed a major perturbation in energy metabolites in tumors. Indeed, energy-related metabolites ATP, ADP, and AMP as well as the NADH cofactor and the resulting NADH/NAD ratio were all consistently decreased in hepatocarcinoma samples compared to adjacent liver tissue samples. These findings suggest an unbalanced utilization of energy metabolites by HCC cells, and that bioenergetic substrates such as ATP become limiting in such tumors. Strikingly, HCC seems devoid of ATP reserves, which is pertinent in the context of metabolic reprogramming as a response to rapid cell proliferation, a highly energy-demanding cellular process. Compared to previous metabolomics analyses performed on murine HCC cells, certain findings within this study overlap with those from cellular metabolomics, such as decreased glycerol-3P, NADH, NAD, and NADP [10]. Inversely, in murine HCC tissues, ATP, ADP/AMP, and NADH/NAD have been previously found to be decreased, opposing the findings within the human cohort [10]. Indeed, comparisons between cell cultures, murine liver tissue, and actual human liver tissue remain challenging, given their completely different natures. Murine hepatocarcinogenesis occurs in a highly regulated and reproductive environment, whereas HCC in patients is a multi-factorial disease occurring in a much less controlled manner.

Moreover, decreased reductive potential, characterized by a pronounced drop in the GSH/GSSG ratio, was the most important feature of the metabolomic signature of HCC tumors per VIP analysis. In fact, GSH was among the most significantly altered metabolites in HCC, its abundance being markedly lower in tumors than in adjacent non-tumoral tissue. The oxidized form of NADPH, NADP, was also significantly lower in HCC. Given that both GSH and NADPH are major agents involved in the control of cellular oxidative stress, and consequently redox homeostasis, these findings suggest that oxidative stress is likely exacerbated in HCC and that it surpasses the reductive capacity of HCC cells. Decreased GSH and NADP have also been reported previously in murine HCC, which only further highlight the possibility that oxidative stress could be a vulnerability of HCC [10].

Given the important findings of the reported metabolomics analyses of HCC compared to adjacent normal liver tissues, considered with previous findings of metabolomics in HCC and cirrhosis, studying paramount changes in metabolism occurring during liver disease and hepatocarcinogenesis is bound to lead to paramount discoveries for improving the clinical management of HCC [26,27]. Together with other LC/MS studies of HCC, the main overlapping metabolites considered as altered pathways in liver tumors include alanine, arginine, lactate, succinate, NADH, and NADP metabolites [26,27]. Likewise, convincing evidence of the molecular analysis suggests metabolic reprogramming in HCC [19,20,21]. As such, collaboration within the research community on HCC, with a multi-omics approach, is fundamental in the identification of holistic metabolism-based HCC classifications.

## 5. Conclusions

In conclusion, this study combining non-targeted and targeted metabolomics has revealed that the metabolite signatures of HCC and adjacent non-tumoral liver are constitutionally distinct. Through non-targeted ^1^H-NMR analysis, we identified that HCC tumors exhibit much greater metabolomic variability than adjacent non-cancerous liver, which can be likely attributed to the high degree of heterogeneity observed in such cancers. Through targeted LC/MS analysis, on the other hand, we specifically identified a number of metabolic intermediates that are found in lower concentrations in HCC tissues, such as ATP and GSH. Overall, these findings are paramount for the global objective to delineate HCC metabolism and pathophysiology. They could ultimately pave the way for the identification of precision biomarkers of this disease as well as, potentially, novel targets for HCC therapeutics.

## Figures and Tables

**Table 1:** Clinical data of patients with HCC liver tumors and sample cryopreservation time. Collected clinical data of patients who participated for metabolomics analyses of HCC and adjacent non-cirrhotic liver. Time to cryopreservation was observed as the time elapsed between tissue resection and liquid nitrogen storage.

	Sexe	Age	Tissue Type	Grade (Edmonson-Steiner)	Sub-Type	Underlying Liver Condition	Time to Cryopreservation (m)
Patient 1	F	52	HCC	2	Trabecular	Normal liver	25
			Non-tumoral				25
Patient 2	M	73	HCC	2	Trabecular	Normal liver	30
			Non-tumoral				32
Patient 3	F	47	HCC	1	Trabecular	Normal liver	28
			Non-tumoral				30
Patient 4	M	57	HCC	2	Trabecular	Normal liver	28
			Non-tumoral				40
Patient 5	M	67	HCC	2	Trabecular	Normal liver	45
			Non-tumoral				47

## Data Availability

The data presented in this study are available in this article.

## Supplementary Materials

The following supporting information can be downloaded at: https://www.mdpi.com/article/10.3390/cancers15123232/s1, Table S1: Cryopreservation times of studied tumor and non-tumoral liver tissue specimens.
