# Supplementary material for: Metabolomics-Guided Identification of a Distinctive Hepatocellular Carcinoma Signature
Source: Cancers (Basel). 2023 Jun 18;15(12):3232. doi: 10.3390/cancers15123232 (PMC10296227; doi:10.3390/cancers15123232)
Supplement: Supplementary file 1 [file cancers-15-03232-s001.zip › cancers-2396461-supplementary.pdf]

Table S1. Cryopreservation times of studied tumor and non-tumoral liver tissue specimens.

	Specimen type	Time to cryopreservation (m)
Patient 1	Tumor	25
	Non-tumoral	25
Patient 2	Tumor	30
	Non-tumoral	32
Patient 3	Tumor	28
	Non-tumoral	30
Patient 4	Tumor	38
	Non-tumoral	40
Patient 5	Tumor	45
	Non-tumoral	47
